# Widespread circulation of Crimean-Congo haemorrhagic fever virus in ticks, Corsica (France), 2024

**DOI:** 10.1016/j.onehlt.2026.101339

**Published:** 2026-01-22

**Authors:** Morena Gasparine, Armand Namekong Fokeng, Shirley Masse, Eva Lopez, Remi Charrel, Xavier de Lamballerie, Alessandra Falchi

**Affiliations:** a Unite des Virus Emergents (UVE : Aix-Marseille Univ, Universita di Corsica, IRD 190, Inserm 1207, IRBA), France; b Centre National de Référence des Arbovirus, Marseille, France

**Keywords:** CCHFV, Ticks, Cattle, Seroprevalence, Molecular detection, Corsica

## Abstract

Crimean-Congo Haemorrhagic Fever is a severe tick-borne viral disease with a high fatality rate. This study aimed to advance the understanding of CCHF virus (CCHFV) in terms of geographical spread and genotypic characterization by investigating its detection in ticks collected from cattle, one year after the first detection of CCHFV in Corsica. From 2024 to 2025, we collected ticks from cattle, with standardised sampling occurring twice per month. Ticks were screened for CCHFV RNA by RTq-PCR. Genome sequencing and phylogenetic analyses were performed. Blood samples from a subset of these cattle were tested for CCHFV antibodies using a commercial enzyme-linked immunosorbent (ELISA) test. Among the 13,577 ticks collected, CCHFV RNA was detected in 61 pools (1.6%) out of the 3803 tested. We identified CCHFV African genotype III in ticks collected from cattle at different sites in northern and southern Corsica. However, two tick strains showed a reassortant profile, with the S and L segments belonging to genotype III and the M segment belonging to genotype I. Data also showed that our strains clustered with strains isolated in African and Western European countries. The overall IgG anti-CCHFV seroprevalence in cattle was 8.44% [95% CI: 6.27% - 11.06%]. This study provides new insights into the spatial and temporal distribution of CCHFV in Corsica and confirms the wider-than-expected distribution and variability of CCHFV in Corsica. Therefore, our findings confirm the genetic variability inside the CCHFV genotypes and their introduction to Corsica from other countries.

## 1 Introduction

Crimean-Congo Haemorrhagic Fever (CCHF) is a life-threatening disease caused by the Crimean-Congo Haemorrhagic Fever Virus (CCHFV), a tick-borne pathogen classified in the genus *Orthonairovirus* within the family *Nairoviridae* [1]. With a case fatality rate ranging from 5% to 30%, CCHF represents a major public health concern, particularly due to its extensive geographic range, endemic in Africa, the Middle East, Southeast Asia and eastern Europe [2]. CCHFV has caused major outbreaks in 10.13039/501100000780European Union (EU)/European Economic Area (EEA) neighbouring regions, principally in the Balkan region, Turkey and Russia [3] and is emerging in western Europe, as supported by the 18 autochthonous cases detected in Spain since 2013 [4] and one recent fatal case in Portugal [5]. Transmission of CCHFV to humans occurs predominantly via bites of *Hyalomma* (*H.*) ticks, which are considered the main vector for the virus. While the primary vector in Europe is *H. marginatum* [6], *H. lusitanicum* ticks have also been found to carry the virus [7]. Although no human cases have been diagnosed in France to date, there is a risk in southern France because of the recent detection of the virus in ticks collected from cattle [8], [9].

Corsica, a French Mediterranean island, has been identified as an area at risk of CCHF emergence due to: i) serological evidence of CCHFV exposure in domestic livestock since 2014 [10], ii) the presence of *H. marginatum* vector, which is now abundant and widespread [11], and iii) the detection of CCHFV RNA (Africa 1 genotype) in ticks collected from cattle in 2023 [8]. A recent seroprevalence study reported initial evidence of low human exposure to CCHFV in Corsica [12] but highlighting a risk of CCHF among the Corsican population, particularly among farmers and slaughterhouse workers [12]. Altogether, these results are consistent with the emergence of CCHFV and with a risk of human exposure to CCHFV in Corsica.

Therefore, this study aimed to advance the understanding of CCHFV in terms of geographical spread and genotypic characterization in Corsica by investigating its detection in ticks collected from cattle during 12 months, one year after the first detection of CCHFV in ticks.

## 2 Methods

### 2.1 Study area, samples collection and processing

The study was conducted in the French Mediterranean island of Corsica. It consists of two administrative departments (northern and southern Corsica) and five districts (Ajaccio, Bastia, Calvi, Corte, Sartène) including 365 municipalities.

From February 2024 to February 2025, we collected ticks from cattle at the two main slaughterhouses of Corsica (in northern and southern Corsica), with standardised sampling occurring twice per month. At each slaughterhouse, ticks were collected from the hides of 30 randomly selected cattle after skinning. In the southern slaughterhouse only, blood samples were also collected during slaughter into EDTA-supplemented tubes. The identification number of each animal was recorded via its ear tag to trace the municipality of origin, using the National Identification Database. Ticks were morphologically identified to the species level whenever possible based on morphological characters, using taxonomic keys [13]. Once identified, ticks were pooled in groups of up to six individuals by host animal, species, sex, and developmental stage. Pools were then stored at −80 °C until further analysis. Whole blood samples were stored at −20 °C pending analysis.

### 2.2 CCHFV detection and sequencing

#### 2.2.1 Extraction

As CCHFV is a biosafety level 4 (BSL-4) pathogen, it should be handled in high-containment settings, which are often not available or affordable for every institution. Therefore, tick and blood samples were subjected to heat inactivation at 56 °C for 2 h [14] prior to shipment for serological and molecular analyses at the Unité des 50 Virus Emergents (France).

Ticks, whether individual or grouped, were homogenized in 1 mL of Minimal Essential Medium solution (containing antibiotics and fungicide) using the TissueLyser II (Qiagen, Hilden, Germany) at a frequency of 30 cycles per second for 3 min. Before nucleic acid extraction, a standardised quantity of MS2 bacteriophages (10 μL per 100 μL of homogenate) was added to each sample to monitor the steps of nucleic acid extraction, reverse transcription, and amplification by RT-qPCR [15]. Total nucleic acids were extracted from 200 μL of tick homogenate and of blood using magnetic beads with the KingFisher Flex™ system and the MagMAX™ Viral/Pathogen Ultra Nucleic Acid Isolation Kit (Thermofisher Scientific), following the manufacturer's instructions. After extraction, the nucleic acids were suspended in 100 μL of the elution buffer and stored at −80 °C.

### 2.3 Genomic detection and sequencing of CCHFV

Ticks' homogenates and animal blood samples were tested for CCHF virus RNA using simplex reverse transcription (RT) quantitative PCRs targeting the large (L) RNA segment [16]. We used the One-Step RT-PCR System Kit (BiotechRabbit) to design 17 CCHFV-specific pairs of primers to amplify the small (S), medium (M), and Large (L) segments (Supplementary Table S1). We sequenced PCR products by using the Oxford Nanopore Technologies' MinION Mk1C sequencer paired with the SQK NBD114.24 kit (New England Biolabs) following the manufacturer's instructions. The sequences generated were assembled using CLC Genomics Workbench 20.0.4 (Qiagen, France). The effective detection of CCHFV genome is strongly supported as both negative and positive controls were used. The PCR systems can distinguish viral genomic RNA from the positive control [17], and the CCHFV sequences obtained were original and unambiguous.

### 2.4 Phylogenetic analysis

Nucleotide sequences were manually checked, aligned, and cleaned by using Mega12 Software [18]. The CCHFV genomes (segment S, M and L) were searched from the contigs using Basic Local Alignment Search Tool (BLAST). The sequences obtained were aligned with other CCHFV strains from Genbank [9], using ClustalW. We determined the best model by using the maximum-likelihood method and performed phylogenetic analyses by using MEGA12 software. The reliability of the branches was assessed using a bootstrap analysis of 1000 replicates. All strains obtained in this study have been deposited in GenBank, with their accession numbers provided in Supplementary Table S2.

### 2.5 Serological analysis

All whole blood samples collected from cattle in the slaughterhouse of southern Corsica, were tested for the presence of total antibodies against CCHFV using the ID Screen® CCHF Double Antigen Multi-species ELISA kit (IDvet, Grabels, France), following the manufacturer's instructions. Briefly, 50 μL of the kit dilution buffer was added to the wells of 96-well microplates pre-coated with recombinant CCHFV nucleoprotein, followed by 30 μL of each test or control sample. The plates were then incubated at 21 °C (±5 °C) for 45 min. After washing, a recombinant nucleoprotein conjugated to horseradish peroxidase was added and incubated for 30 min. Another washing step was performed before adding the substrate solution. Finally, the reaction was stopped and the optical density (OD) was measured at 450 nm using a plate reader (Thermo Scientific™ Multiskan™, Waltham, MA, USA). According to the manufacturer, a test run is valid if the optical density measured at 450 nm (OD450 nm) of the positive control (ODPC) is greater than 0.35 and the ratio of the ODPC to the OD450 nm of the negative control (ODNC) is greater than 3. Interpretation of tested samples is based on the ratio of the sample OD450 nm (ODS) to ODPC expressed as percentage: (ODS / ODPC) x 100 (hereafter S/P (%)). A sample was classified as reactive if S/P (%) was over 30%. Serum samples with S/P (%) below or equal to 30% were considered as non-reactive.

### 2.6 Statistical analysis

All data were entered, cleaned and validated in MS Office Excel 2016 and then analysed using R software (R Core Team), version 4.2.2. Characteristics of cattle (age, sex and location) and of ticks (species, sex and stages) were summarized using frequencies and percentages, first overall and then stratified by study groups. To investigate the association between tick infestation rate (TIR) and individual characteristics (age and sex) as well as the municipality of origin of the cattle, generalized linear models (GLMs) with a log link function were fitted.

A Poisson model including age, sex, and municipality showed pronounced overdispersion (Pearson dispersion parameter ≈ 22.7), indicating a violation of Poisson model assumptions. Therefore, statistical inference was based on a quasi-Poisson model. In addition, complementary non-parametric analyses were conducted to support the GLM findings, including a Spearman rank correlation test to assess the relationship between age and TIR, a Wilcoxon test to evaluate the association between sex and TIR, and an analysis of variance (ANOVA) to examine differences in TIR among municipalities. Seroprevalence for CCHFV was determined for each sampling site, age group and sex using the proportion of positive cases relative to the total number of samples in each subgroup. The 95% confidence intervals (CIs) were calculated for proportions using a binomial distribution. Maps showing district of livestock origin, CCHFV RNA detection spots, seroprevalence values and *H. marginatum* repartition were constructed using plotted Quantum GIS (QGIS) version 3.16.0. A Voronoi diagram where polygon centroids represent the municipalities of origin of the sampled cattle was applied to illustrate seroprevalence.

## 3 Results

### 3.1 Tick collection and identification

In total, 13577 ticks were collected from 1096 cattle. Ticks were grouped into 3803 pools: 2390 (62.9%) pools including 8545 (62.9%) ticks from southern Corsica, 1368 (35.9%) pools containing 4835 (35.6%) ticks from northern Corsica. A further 197 ticks could not be identified from both northern and southern Corsica were grouped into 45 pools (Table 1). Among the 1096 cattle, originating from 134 municipalities across the island, 61.5% (*n* = 674) were males, with a median age of 7 months [min =2 months; max = 28.08 years]. Among them, 68.8% (*n* = 754) were infested with at least one tick (Table 2).

**Table 1:** : Description of main characteristics of ticks collected from cattle natives of municipalities of Northern and Southern Corsica.

Characteristics	Northern Corsica^*1*^	Southern Corsica^*1*^	NA⁎^*1*^	Overall^*1*^
**Ticks**^***1***^				
*Overall*	4835 (35.6)	8545 (62.9)	197 (1.5)	13,577 (100.0)
**Tick species**^***1***^				
*Rhipicephalus bursa*	2413 (49.9)	4452 (52.1)	170 (86.3)	7035 (51.8)
*Hyalomma marginatum*	483 (10.0)	1340 (15.7)	20 (10.2)	1843 (13.6)
*Hyalomma scupense*	584 (12.1)	1044 (12.2)	2 (1.0)	1630 (12.0)
*Rhipicephalus* spp.	292 (6.0)	822 (9.6)	1 (0.5)	1115 (8.2)
*Boophilus annulatus*	676 (14.0)	10 (0.1)		686 (5.1)
*Rhipicephalus sanguineus*	118 (2.4)	290 (3.4)	1 (0.5)	409 (3.0)
*Ixodes ricinus*	53 (1.1)	265 (3.1)	3 (1.5)	321 (2.4)
*Haemaphysalis punctata*	125 (2.6)	161 (1.9)		286 (2.1)
*Hyalomma* spp.	82 (1.7)	115 (1.3)		197 (1.5)
*Dermacentor marginatus*	6 (0.1)	43 (0.5)		49 (0.4)
*NA*	3 (0.1)	3 (0.04)		6 (0.04)
**Sex**^***1***^				
*M*	2630 (54.4)	4906 (57.4)	146 (74.1)	7682 (56.6)
*F*	1680 (34.7)	2609 (30.5)	51 (25.9)	4340 (32.0)
*NA*	525 (10.9)	1030 (12.1)		1555 (11.5)
**Stages**^***1***^				
*Larva*	2 (0.04)	17 (0.2)		19 (0.1)
*Nymph*	522 (10.8)	1010 (11.8)		1532 (11.3)
*Adult*	4311 (89.2)	7518 (88.0)	197 (100.0)	12,026 (88.6)
^*1*^*n (%)*				

⁎ Ticks collected from cattle not identified.

**Table 2:** : Demographic characteristics of cattle natives of municipalities of Northern and Southern Corsica.

Characteristics	Northern Corsica^1^	Southern Corsica^1^	NA^1^	Overall^1^
**Cattle**^***1***^				
*Overall*	572 (52.2)	513 (46.8)	11 (1.0)	1096 (100.0)
**Age**^***1***^				
*Median (months); min-max*	8; 2–337	7; 3–276		7; 2–337
*2–12 months*	465 (81.3)	407 (79.3)		872 (79.6)
*1–2 years*	57 (10.0)	58 (11.3)		115 (10.5)
*2–5 years*	21 (3.7)	22 (4.3)		43 (3.9)
*5–30 years*	29 (5.1)	26 (5.1)		55 (5.0)
*NA*			11 (100.0)	11 (1.0)
**Sex**^***1***^				
*M*	352 (61.5)	322 (62.8)		674 (61.5)
*F*	220 (38.5)	191 (37.2)		411 (37.5)
*NA*			11 (100.0)	11 (1.0)
**Tick collected**^***1***^				
*Yes*	336 (58.7)	407 (79.3)	11 (100.0)	754 (68.8)
*No*	236 (41.3)	106 (20.7)		342 (31.2)
^*1*^*n (%)*				

The morphological identification of ticks revealed the presence of five genera of Ixodid ticks, *Rhipicephalus*, *Hyalomma*, *Ixodes*, *Haemaphysalis* and *Dermacentor*. We identified eight different tick species, including 7035 (51.8%) *Rhipicephalus (R.) bursa*, 1843 (13.6%) *H. marginatum*, 1630 (12.0%) *H. scupense*, 686 (5.1%) *R. Boophilus annulatus*, 409 (3.0%) *R. sanguineus*, 321 (2.4%) *Ixodes ricinus*, 286 (2.1%) *Haemaphysalis (Ha.) punctata* and 49 (0.4%) *Dermacentor marginatus*. A total of 1312 ticks have been identified to the genus level, including 1115 (8.2%) belonging to *Rhipicephalus* spp. and 197 (1.5%) to *Hyalomma* spp. which were at nymph stage. Six ticks (0.04%) could not be identified due to collection damages but have been analysed (Table 1).

### 3.2 Molecular detection of CCHFV in ticks

**Fig. 1:**
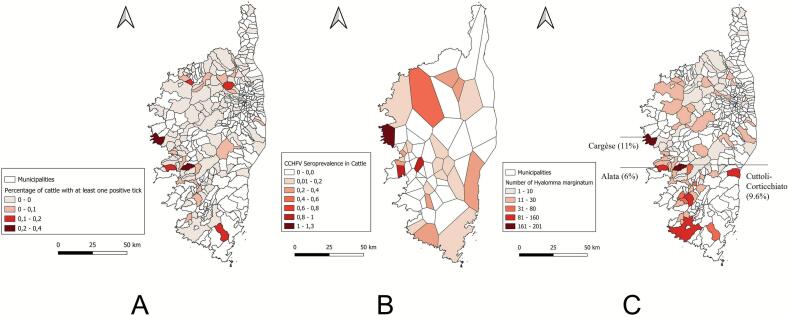
Maps illustrating A) the spatial distribution of cattle with at least one tick testing positive; B) the prevalence of IgG against CCHFV, displayed using a Voronoi diagram where polygon centroids represent the municipalities of origin of the sampled cattle; C) the distribution of *Hyalomma marginatum* collected during the 2024/2025 period.

Sixty one out of the 3803 tick pools were positive by the L-RNA assay, representing 269 ticks collected from 31 cattle (Table 3) aged between three months and 19 years, and comprising 20 males and 11 females. Of the 31 cattle tested, one had 100% (*n* = 2) of its pools testing positive for CCHFV L-RNA, while for the other 30, the proportion of positive pools ranged from 3.2% to 50%. *R. bursa* was the most prevalent tick species in the majority of positive pools (34 pools, 55.7%), followed by *H. marginatum* (18 pools, 30.0%). *H. scupense* (5 pools, 8.1%), *Rhipicephalus* spp. (2 pools, 3.3%) and *Ha. punctata* (1.6%). Nineteen (61.3%) of the 31 cattle from which CCHFV L-RNA positive ticks were collected, grazed in municipalities of the central-western area, while the others originated from municipalities in the north (Fig. 1-A).

**Table 3:** : Individual data for cattle with CCHFV-positive tick pools collected at slaughterhouses.

Cattle ID	Pool ID	Slaughter-house	Sampling date	Cattle age (months)	Cattle sex	Municipality	Number of pools collected by cow n (% of positive pools)	Tick number by pool CCHFV positive	Tick species	Tick sex	Tick stage	Ct values L assay
FR2030012791	590	Cuttoli	16/4/2024	138	F	Cuttoli-Corticchiato	3 / 20 (15%)	6	*Hyalomma scupense*	M	Adult	32
	593	Cuttoli	16/4/2024	138	F	Cuttoli-Corticchiato		4	*Hyalomma marginatum*	F	Adult	28
	594	Cuttoli	16/4/2024	138	F	Cuttoli-Corticchiato		1	*Hyalomma scupense*	M	Adult	22
FR2005373926	743	Cuttoli	30/4/2024	7	M	Canavaggia	1 / 2 (50%)	1	*Rhipicephalus bursa*	M	Adult	IND
FR2005373923	751	Cuttoli	30/4/2024	9	M	Canavaggia	1 / 3 (33.3%)	1	*Rhipicephalus bursa*	M	Adult	IND
FR2030890747	761	Cuttoli	30/4/2024	7	F	Casaglione	1 / 8 (12.5%	2	*Rhipicephalus bursa*	M	Adult	38
FR2030897431	1002	Cuttoli	14/5/2024	7	F	Alata	8 (37.5%)	4	*Hyalomma scupense*	M	Adult	26
	1003	Cuttoli	14/5/2024	7	F	Alata		3	*Hyalomma marginatum*	M	Adult	26
	1008	Cuttoli	14/5/2024	7	F	Alata		1	*Hyalomma marginatum*	M	Adult	28
FR2030895494	1070	Cuttoli	14/5/2024	11	M	Zigliara	3 / 12 (25%)	4	*Hyalomma marginatum*	F	Adult	IND
	1072	Cuttoli	14/5/2024	11	M	Zigliara		1	*Hyalomma scupense*	F	Adult	IND
	1073	Cuttoli	14/5/2024	11	M	Zigliara		1	*Hyalomma marginatum*	F	Adult	IND
FR2030895236	1123	Cuttoli	14/5/2024	12	M	Grosseto-Prugna	1 / 7 (14.3%)	5	*Rhipicephalus bursa*	F	Adult	IND
FR2030894850	1163	Cuttoli	14/5/2024	7	M	Tolla	1 / 6 (16.6%)	1	*Rhipicephalus bursa*	M	Adult	IND
FR2030897440	1414	Cuttoli	11/6/2024	7	M	Alata	1 / 8 (12.5%)	6	*Rhipicephalus bursa*	M	Adult	32
FR2030897015	1501	Cuttoli	11/6/2024	7	M	Sotta	3/22 (13.6%)	6	*Rhipicephalus bursa*	M	Adult	IND
	1508	Cuttoli	11/6/2024	7	M	Sotta		2	*Hyalomma marginatum*	M	Adult	IND
	1510	Cuttoli	11/6/2024	7	M	Sotta		6	*Rhipicephalus bursa*	F	Adult	IND
FR2030897020	1537	Cuttoli	11/6/2024	6	M	Sotta	1/19 (5.26%)	6	*Rhipicephalus bursa*	M	Adult	36
FR2005367430	1768	Ponte Leccia	11/6/2024	8	F	Pieve	1 / 3 (33.3%)	2	*Hyalomma scupense*	M	Adult	IND
FR2005279924	1772	Ponte Leccia	11/6/2024	96	F	Pietralba	1 / 6 (16.6%)	6	*Rhipicephalus bursa*	M	Adult	IND
FR2030901157	1839	Cuttoli	25/6/2024	7	M	Propriano	1 / 2 (50%)	6	*Rhipicephalus bursa*	M	Adult	IND
	1847	Cuttoli	25/6/2024	7	M	Propriano		6	*Rhipicephalus bursa*	M	Adult	34
FR2030710211	1917	Cuttoli	25/6/2024	229	F	Cargese	1/14 (7.1%)	6	*Rhipicephalus bursa*	M	Adult	IND
FR2030895391	2005	Cuttoli	25/6/2024	5	M	Petreto-Bicchisano	1 /10 (10%)	6	*Rhipicephalus bursa*	M	Adult	IND
FR2030893061	2060	Cuttoli	25/6/2024	4	M	Cuttoli-Corticchiato	1/31 (3.2%)	5	*Rhipicephalus bursa*	F	Adult	IND
FR2030893059	2150	Cuttoli	25/06/2024	14	M	Cuttoli-Corticchiato	2 / 33 (6.1%)	6	*Hyalomma marginatum*	M	Adult	IND
	2151	Cuttoli	25/06/2024	14	M	Cuttoli-Corticchiato		5	*Hyalomma marginatum*	M	Adult	32
FR2005350859	2160	Ponte Leccia	25/06/2024	35	F	Avapessa	1 / 7 (14.3%)	1	*Hyalomma scupense*	M	Adult	IND
FR2005374115	2185	Ponte Leccia	25/06/2024	4	M	Canale di Verde	2 / 29 (6.8%)	6	*Rhipicephalus bursa*	M	Adult	IND
	2196	Ponte Leccia	25/06/2024	4	M	Canale di Verde		1	*Hyalomma marginatum*	M	Adult	IND
FR2005375228	2238	Ponte Leccia	25/06/2024	6	F	Zilia	1 / 11 (9%)	6	*Rhipicephalus bursa*	F	Adult	IND
FR2005375229	2247	Ponte Leccia	25/06/2024	6	M	Zilia	1 / 21 (4.7%)	6	*Rhipicephalus bursa*	M	Adult	IND
FR2005360343	2269	Ponte Leccia	25/06/2024	7	F	Ghisoni	1 / 2 (50%)	5	*Rhipicephalus bursa*	F	Adult	IND
FR2005368079	2270	Ponte Leccia	25/06/2024	12	M	Valle-di Rostino	1 / 6 (16.6%)	2	*Hyalomma marginatum*	M	Adult	IND
FR2005349372	2284	Ponte Leccia	25/06/2024	6	M	Montegrosso	1 / 9 (11.1%)	3	*Rhipicephalus bursa*	F	Adult	IND
FR2030891844	2308	Cuttoli	09/07/2024	13	F	Cargese	1 / 5 (20%)	3	*Rhipicephalus bursa*	M	Adult	IND
FR2030900635	2425	Cuttoli	09/07/2024	4	M	Cuttoli-Corticchiato	56 (33.9%)	6	*Rhipicephalus bursa*	M	Adult	34
	2428	Cuttoli	09/07/2024	4	M	Cuttoli-Corticchiato		6	*Rhipicephalus bursa*	M	Adult	36
	2432	Cuttoli	09/07/2024	4	M	Cuttoli-Corticchiato		6	*Rhipicephalus bursa*	M	Adult	32
	2436	Cuttoli	09/07/2024	4	M	Cuttoli-Corticchiato		6	*Rhipicephalus bursa*	M	Adult	36
	2437	Cuttoli	09/07/2024	4	M	Cuttoli-Corticchiato		6	*Rhipicephalus bursa*	M	Adult	36
	2438	Cuttoli	09/07/2024	4	M	Cuttoli-Corticchiato		6	*Rhipicephalus bursa*	M	Adult	34
	2439	Cuttoli	09/07/2024	4	M	Cuttoli-Corticchiato		6	*Rhipicephalus bursa*	M	Adult	34
	2440	Cuttoli	09/07/2024	4	M	Cuttoli-Corticchiato		6	*Rhipicephalus bursa*	M	Adult	32
	2447	Cuttoli	09/07/2024	4	M	Cuttoli-Corticchiato		6	*Rhipicephalus bursa*	F	Adult	32
	2448	Cuttoli	09/07/2024	4	M	Cuttoli-Corticchiato		6	*Rhipicephalus bursa*	F	Adult	32
	2449	Cuttoli	09/07/2024	4	M	Cuttoli-Corticchiato		6	*Rhipicephalus bursa*	F	Adult	36
	2450	Cuttoli	09/07/2024	4	M	Cuttoli-Corticchiato		6	*Rhipicephalus bursa*	F	Adult	32
	2452	Cuttoli	09/07/2024	4	M	Cuttoli-Corticchiato		6	*Rhipicephalus bursa*	F	Adult	34
	2455	Cuttoli	09/07/2024	4	M	Cuttoli-Corticchiato		6	*Hyalomma marginatum*	M	Adult	30
	2456	Cuttoli	09/07/2024	4	M	Cuttoli-Corticchiato		6	*Hyalomma marginatum*	M	Adult	20
	2457	Cuttoli	09/07/2024	4	M	Cuttoli-Corticchiato		5	*Hyalomma marginatum*	M	Adult	30
	2458	Cuttoli	09/07/2024	4	M	Cuttoli-Corticchiato		6	*Hyalomma marginatum*	F	Adult	28
	2459	Cuttoli	09/07/2024	4	M	Cuttoli-Corticchiato		6	*Hyalomma marginatum*	F	Adult	30
	2460	Cuttoli	09/07/2024	4	M	Cuttoli-Corticchiato		4	*Hyalomma marginatum*	F	Adult	30
FR2030902787	3154	Cuttoli	22/10/2024	6	F	Cargèse	2 / 2 (100%)	1	*Hyalomma marginatum*	M	Adult	18
	3155	Cuttoli	22/10/2024	6	F	Cargèse		1	*Hyalomma marginatum*	F	Adult	34
FR2030873569	3424	Cuttoli	10/12/2024	33	M	Sarrola-Carcopino	16 (6.25%)	6	*Rhipicephalus* spp.	NA	Nymph	36
FR2030906654	3445	Cuttoli	10/12/2024	7	M	Cargèse	6 (16.6%)	6	*Rhipicephalus* spp.	NA	Nymph	30
FR2005384138	3500	Ponte Leccia	10/12/2024	3	M	Olmi-Cappella	8 (12.5%)	2	*Haemaphisalis punctata*	F	Adult	28

IND: Undetermined.

**Fig. 2:**
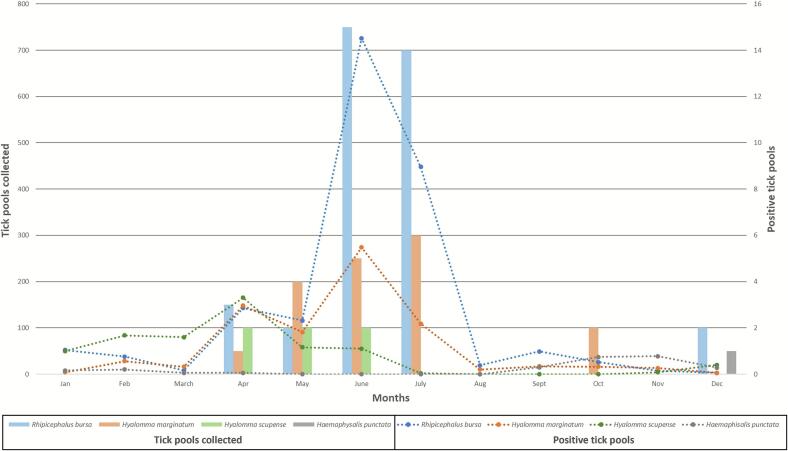
Comparison between tick abundance and CCHFV positivity in the four tick species found positive.

Positive ticks were collected between April and December 2024, with 56 pools (91.8%) collected between April and August (Fig. 2). Five pools (8.2%) were collected from October to December 2024. Two of these five pools were constituted by two adults of *H. marginatum*, two by 12 nymphs of *Rhipicephalus* spp. and one by two adults of *Ha. punctata* (Table 3).

### 3.3 CCHFV seroprevalence in cattle

Blood samples were collected from 557 out of the 1096 cattle included in the southern slaughterhouse (Supplementary Table 3). Although all animals were slaughtered in southern Corsica, 80% (*n* = 444) originated from 47 municipalities in southern Corsica, while 20% (*n* = 111) came from 15 farms located in northern Corsica. Among the 557 cattle, 61.4% (*n* = 342) were males. The median age of enrolled cattle was 7 months [2 months-8.4 years].

The overall seroprevalence of CCHFV antibodies was 8.4% (*n* = 47 out of 557; 95% CI: 6.27–11.06). 80.9% (*n* = 38) of the seropositive animals were 1 year old or younger and 55.3% (*n* = 26) were males (Supplementary Table S3). Given the large number of municipalities sampled (*n* = 62) and the heterogeneous geographical distribution of seroprevalence, the municipalities were grouped into the main five districts (Supplementary Table S3). CCHFV seropositivity ranged from 0% in Bastia to 66% in Ajaccio. Two out of the 31 cattle (ID FR2030902787 and FR2030906654 referenced in Table 3) that tested positive for CCHFV L-RNA positive ticks showed anti-CCHFV IgG antibodies, and both originated from the same municipality in the central-western area of Corsica (Cargèse). CCHFV seropositivity and CCHFV L-RNA ticks' positivity were not associated with the livestock declaration district (*p*-value = 0.5 and 0.3, respectively). Neither the age nor the sex of the cattle was correlated with being seropositive or having CCHFV L-RNA positive ticks positive ticks (Supplementary Table S3 and S4).

### 3.4 Phylogenetic analysis

Phylogenetic analysis was performed on 14 CCHFV-positive tick pools using the S segment, which is commonly employed for genotyping and phylogenetic studies due to its sufficient genetic diversity to distinguish viral strains while remaining relatively conserved. This segment enables the classification of CCHFV into distinct clades, often associated with their geographic origins [19]. All analysed S segments belonged to genotype III, which has been reported in central and southern Africa as well as in Spain and southern France [9], [20], [21], [22].

**Fig. 3:**
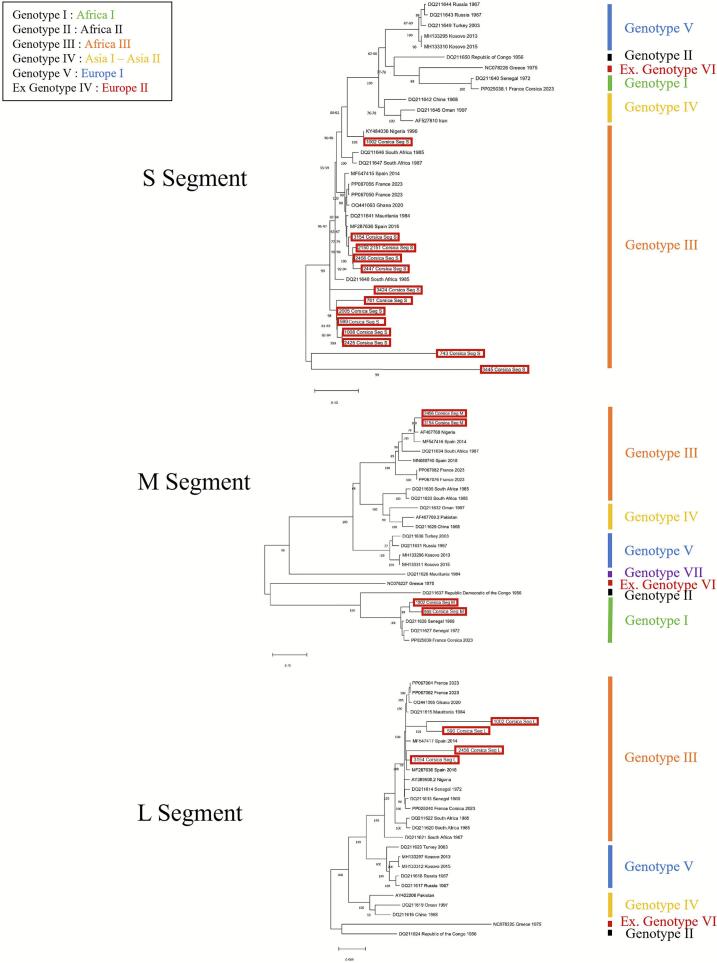
Phylogenetic trees of CCHFV S, M and L segment based on 1671 nt, 5413 nt and 12,040 nt respectively. Trees were generated by the Maximum-Likelihood method (ML) with General Time Reversible model. Bootstrap confidence limits were based on 1000 replicates. Scale bars indicate nucleotide substitutions per site.

Among the 14 samples analysed, one (7.14%) was identical to a previously described Nigerian strain; six (42.86%) were closely related to strains circulating in Spain and southern France, although only two showed specific similarity to strains from southern France; and seven (50%) clustered with a South African strain. Phylogenetic trees grouped the sequences into six distinct genotypes (Fig. 3).

Complete genome sequences were obtained for four of the 14 tick pools. Sample selection was based on the availability of at least one pool per bovine and on low Ct values, which ranged from 18 to 32 (Table 3). Consequently, complete genomes were recovered only from the four pools with the lowest Ct values. While all four strains belonged to genotype III based on the S and L segments, two strains clustered with genotype I based on the M segment. These two strains were identified in pools 590 and 1002 of *Hyalomma scupense* collected from two cattle originating from central-western Corsica (Table 3; Fig. 3).

#### 3.4.1 S segment

All samples analysed in this study (*n* = 14) clustered within genotype III based on S segment analysis (Fig. 3). Sample 1002 showed 100% nucleotide identity with a strain detected in Nigeria in 1996 (KY484036). Samples 2456, 3154, and 2150_2151 were closed and exhibited nucleotide similarities ranging from 97.45% to 98.86% with CCHFV S fragments from Spain (MF287636) and southern France (PP067050 and PP067055). The 2005 strain shared 95.45% nucleotide similarity with a 1985 South African strain (DQ211648).

#### 3.4.2 M segment

The M segment of samples 2456 and 3154 were identical, showing 96.86% nucleotide similarity with a CCHFV isolate from Nigeria (AF467768), classified within Genotype III (Fig. 3). In contrast, samples 1002 and 590 shared respectively 95.48% and 93.43% nucleotide identity with a CCHFV strain detected in Senegal in 1969 (DQ211626), belonging to Genotype I (Fig. 3).

#### 3.4.3 L segment

The L segment of samples 2456 and 3154 showed 91.67% and 98.47% nucleotide similarity, respectively, with a CCHFV strain identified in Spain in 2016 (MF287638). Sample 590, exhibited 95.66% homology with strains collected in southern France in 2023 (PP067050 and PP067055), and shared 94.90% nucleotide similarity with sample 1002. All four samples clustered within genotype III (Fig. 3).

### 3.5 Correlation between TIR and cattle geodemographic variables

Host age was positively associated with TIR in the quasi-Poisson model (β = 0.00767, SE = 0.00105, *p* = 5.7 × 10^−13^). This corresponds to an incidence rate ratio (IRR) of 1.0077 per month (95% CI: 1.0056–1.0098), indicating that each additional month of age was associated with an estimated 0.77% increase in tick infestation rate. In contrast, a non-parametric rank correlation test did not detect a monotonic association between TIR and age (Spearman's ρ = 0.026, *p* = 0.39).

After adjustment for age and municipality, male cattle exhibited significantly higher TIR than females (β = 0.436, SE = 0.101, *p* = 1.7 × 10^−5^). This corresponds to an IRR of 1.55 (95% CI: 1.27–1.89), indicating that males had an estimated 55% higher tick infestation rate than females. Unadjusted comparisons between sexes did not reveal significant differences (Wilcoxon test *p* = 0.66).

Tick infestation rates varied markedly across municipalities. The variance analysis showed a highly significant municipality effect (*p* < 2 × 10^−16^), and the multivariable GLM confirmed strong spatial heterogeneity in TIR.

## 4 Discussion

The present study provides new insights into the spatial and temporal distribution of CCHFV in Corsica and confirm the wider-than-expected distribution and variability of CCHFV in Corsica. These findings are compatible with reports of the genetic variability of the virus, because CCHFV is well known to undergo reassortment to produce diverse combinations of the S, L, and M segments. The present investigation of CCHFV detection in ticks collected from cattle, supports the wide endemicity of CCHFV in Corsica, as reported in new areas with respect to those described in 2023, with a main circulation of strains belonging to genotype III whereas genotype I was described in 2023 [8].

In this study two *Hyalomma* species have been identified, *H. marginatum* and *H. scupense*, which accounted for nearly 10% of ticks collected from cattle. *H. marginatum* remained predominantly concentrated in municipalities where CCHFV L-RNA was detected. Specifically, 11% of them were found in the central west of the island confirming the main role of this tick in the maintenance of the virus in livestock populations and in the surrounding environment (Fig. 1-C). *R. bursa*, due to its predominance during the active season of *H. marginatum*, could also play a role in the virus's circulation. Several experimental studies have suggested that *Rhipicephalus* species play a role in CCHFV transmission [23]. In the case of *R. bursa*, CCHFV has been found in unfed ticks, which provides unequivocal evidence that the virus survives tick moulting and is transmitted transovarially from engorged females to larvae via eggs [24]. Furthermore, *R. bursa* ticks appear to be the primary vector of CCHFV genotype VI (Europe 2), which has been reclassified as Aigai virus, in Balkan countries [25]. Nevertheless, the detection of the virus during the winter period — when *H. marginatum* is less active — suggests that other tick species may also contribute to the transmission of CCHFV (Fig. 2), notably *Ha. punctata*, which pools have been yet detected positive by the CCHFV L-RNA assay at the end of 2023 September in the south west of Corsica [8]. CCHFV has been found in *Ha. punctata* collected from ungulates on several occasions [26]. Recently, a study in southern Russia detected CCHFV in one unfed *Ha. punctata* specimen out of 477 examined [27]. These findings suggest that *Ha. punctata* may also act as a vector for CCHFV, although its role in maintaining the virus remains unclear.

The proportion of tick pools collected from cattle and containing at least one CCHFV-positive tick nearly tripled in 2024 compared with the 2023 data from a previous study, increasing from 1% (5/465) to 2.8% (31/1096) [8]. Viral RNA was detected locally in the central-western part of the island, specifically in municipalities with the highest seroprevalence rates estimated in the present study (Cargèse: 1.26%; Alata and Cuttoli-Corticchiato: 0.90% as show in Supplementary Table S5 and Fig. 1-B) which also correspond to municipalities where *H. marginatum* was abundantly collected (Fig. 1-C). This area had already reported viral detection in four animals sampled in the southern slaughterhouse during 2023 [8]. In this study, for the first time, CCHFV RNA was detected in municipalities in northern Corsica, where seroprevalence rates exceeded 50% in cattle sera sampled during 2014–2016 [10].

The lack of a strong association between age and tick infestation rate (TIR) in the Spearman analysis likely reflects the discrete and heterogeneous nature of the count data, highlighting the limitations of rank-based tests. Similarly, the absence of a significant sex effect in the Wilcoxon test suggests that sex related differences in TIR become apparent only after accounting for confounding by age and geographic location. Although municipality specific effects were often not individually significant under quasi Poisson inference, likely due to limited precision in sparsely sampled areas, the overall results consistently indicate that geographic location is a determinant of observed tick infestation. However, this spatial pattern may partly reflect unaccounted temporal variation, as the season of sample collection was not included in the analyses and may contribute to differences observed across municipalities.

Analysis of the L segment in 4 out of the 13 positive samples revealed that they also belonged to genotype III. Phylogenetic analyses generally show a strong concordance between genotypes defined by the L and S segment, highlighting a geographical structuring of the virus [28].

Furthermore, the reassortment observed in two of the four sequenced M segments suggests a co-circulation of two strains in the region, or at least evidence of past co-circulation, given the current predominance of genotype III in 2024. This phenomenon likely occurred in ticks, where co-infections are more probable due to their long lifespan and capacity to acquire multiple viral strains, making them key hosts for reassortment events [28]. As the result was obtained from pooled ticks, it cannot be determined with certainty whether this represents a reassortment event or the presence of multiple ticks infected with different strains within the pool. However, the quality of the obtained sequence indicates that it may more likely represent a reassortment event.

The geographical distribution of the strains, based on segments S and L, suggests that the introduction of this strain may have occurred via migratory birds. Although birds are not considered amplifying hosts of the virus [29], they could nevertheless act as mechanical vectors, contributing to the spread of the virus to new geographic areas. This hypothesis had already been proposed during the detection of genotype I in 2023 [8]. However, the current detection of genotype III suggests that Corsica is not located along a single avian migratory route, but rather at the intersection of several, which may have facilitated the introduction of distinct strains. It is also possible that genotype III was introduced from Senegal, where both genotypes I and III have been detected [30]. The genotype I strain identified in 2023 was genetically similar to those found in Senegal, and suggests that a single avian migratory route may have been sufficient to introduce the virus into the island. However, analysis of the S segment from samples in this study shows greater genetic similarity with sequences from other regions, particularly Nigeria, South Africa, and Spain/southern France, which may call this latter hypothesis into question.

In parallel, whole cattle blood from the southern slaughterhouse has also been collected and serological analyses for the detection of IgG against CCHFV were carried out. The overall seroprevalence among cattle was 8.44%. However, as this study mainly includes cattle from the southern half of Corsica, this rate is probably underestimated, especially since the highest seroprevalence rates estimated by Grech-Angelini et al. were found in northern Corsica [10]. Further research is needed to establish a more accurate and representative seroprevalence rate that reflects the situation across the entire island. Other Mediterranean countries have reported cattle seroprevalence rates comparable to the one estimated in this study: 1.89% in Southern Italy [31], 7% in Albania [32] and Central Macedonia, Greece [33], 11.1% in Tunisia [34], 14.97% in Bosnia and Herzegovina [35], and 23.3% in Turkey [36].

Most seroprevalence studies on CCHFV identify age as a major risk factor, as older cattle are more likely to have been exposed to ticks and, consequently, to the virus [37], [38]. However, in the present study, this expected correlation was not observed, likely due to the age distribution of the sampled animals. In particular, the proportion of young cattle (≤1 year old) slaughtered for meat was considerably higher than older animals.

While this study provides valuable insights, several limitations should be acknowledged. First, a large proportion of the samples showed high Ct values for virus detection (Table 3), which limited the recovery of usable genome sequences. Although most of these samples were excluded from the phylogenetic analyses, some were still included in the phylogenetic tree construction despite containing only 335 nucleotides for the S segment. This represents a relatively small portion of the viral genome and should be taken into account. We also noticed that, unlike in 2023, very few cattle in this study originated from the south-eastern part of the island, and no tick sample was detected positive in that area. Further investigations at the local slaughterhouse in the southeast (Porto-Vecchio) could be valuable to determine whether the virus is still circulating in this region. Detection of CCHFV in engorged ticks reflects virus circulation within the cattle population but does not provide any evidence regarding the role of ticks—or any specific tick species—in CCHF transmission, as the ticks were feeding on potentially infected cattle. Taken together these results suggest that central-western Corsica, represents a high-risk area for CCHF emergence. Consequently, it is crucial to improve the epidemiological and zoological surveillance of the CCHFV using a sentinel approach to better understand its transmission dynamics and mitigate the risk of emergence.

## 5 Conclusion

This study provides evidence of the increasing detection of CCHFV in Corsica, through the combined detection of viral RNA in ticks and seroprevalence in cattle. CCHFV RNA was detected in multiple tick species, with *R. bursa* and *H. marginatum* being the most frequently associated with positive pools, suggesting their potential involvement in the local transmission cycle. The seasonal and spatial distribution of infected ticks indicates ongoing viral activity across several municipalities, notably in central-western Corsica, where both high seroprevalence and high tick infection rates were observed. Viral RNA was also detected for the first time in ticks from northern Corsica. Phylogenetic analyses based on the S, M, and L genome segments confirmed that all sequences clustered within genotype III, genetically related to strains from Spain, Nigeria, South Africa, and southern France. The detection of reassorted M segment sequences further suggests past or current co-circulation of different viral strains on the island. Together, these findings support the hypothesis of multiple introductions of the virus into Corsica, potentially via migratory birds, and highlight the island's position at the intersection of distinct ecological and migratory pathways. The observed increase in the number of cattle hosting CCHFV-infected ticks reveals an intensification of viral circulation, emphasising the importance of continuing to monitor and refine our understanding of the virus's epidemiological dynamics.

## Use of artificial intelligence tools

None declared.

## Preprint

None declared.

## CRediT authorship contribution statement

**Morena Gasparine:** Writing – review & editing, Writing – original draft, Visualization, Project administration, Methodology, Investigation, Formal analysis, Data curation, Conceptualization. **Armand Namekong Fokeng:** Writing – review & editing, Methodology, Investigation. **Shirley Masse:** Writing – review & editing, Methodology. **Eva Lopez:** Writing – review & editing, Methodology, Investigation. **Remi Charrel:** Writing – review & editing, Validation, Supervision. **Xavier de Lamballerie:** Writing – review & editing, Validation, Supervision. **Alessandra Falchi:** Writing – review & editing, Writing – original draft, Validation, Supervision, Project administration, Methodology, Funding acquisition, Formal analysis, Conceptualization.

## Ethical statement

Ethical approval was not required for this study as it was conducted as part of routine surveillance activities and did not involve the collection of personal or sensitive data. The study complied with applicable national regulations on public health surveillance.

## Funding statement

This work has benefited from a government grant managed by the 10.13039/501100001665Agence Nationale de la Recherche under the Plan France 2030 entitled 10.13039/100010238ARCHE, ANR-23-PEPZ-0003. It also was supported in part by the EVORA (European Viral Outbreak Response Alliance) project (call HORIZON-INFRA-2023-DEV-01-04). The material was provided by the European virus archive-Marseille (EVAM) under the label technological platforms of 10.13039/100007586Aix-Marseille University.

## Declaration of competing interest

None declared.

## Data Availability

The original data generated in this study are publicly available under the accession numbers listed in Supplementary Table S2, and can be accessed via GenBank at: https://www.ncbi.nlm.nih.gov/genbank/.

## Appendix A Supplementary data

Supplementary material Image 1
