# Supplementary material for: Widespread circulation of Crimean-Congo haemorrhagic fever virus in ticks, Corsica (France), 2024
Source: One Health. 2026 Jan 22;22:101339. doi: 10.1016/j.onehlt.2026.101339 (PMC12860254; doi:10.1016/j.onehlt.2026.101339)
Supplement: Supplementary file 1 — Supplementary material [file mmc1.docx]

Supplementary Table S1 : Primer's sytems used to sequenced CCHFV whole genome

| Segment | Primer's ID | Primer | System's name | Lenght (nt) |
| --- | --- | --- | --- | --- |
| S Segment | GP_CCHF_seg S_1S | TCTCAAAGAAACACGTGCCGC | Couple 1 | 530 |
|  | GP_CCHF_seg S_21S | CTTACGCCCACAGTGTTCTC |  |  |
|  | GP_CCHF_seg S_530R | GACATRACAATYTCRCCAGG |  |  |
|  | GP_CCHF_seg S_452S | GTGTCAATGCAAATACGGCAG | Couple 2 | 441 |
|  | GP_CCHF_seg S_893R | GGTTTGTTATCATGYTGTCRGC |  |  |
|  | GP_CCHF_seg S_778S | GCCTTGCCAAGCTTGCAGAG | Couple 3 | 291 |
|  | GP_CCHF_seg S_1069R | GGGTGCTTAAGAGTGCCTTC |  |  |
|  | GP_CCHF_seg S_1030S | CGAACTGGGGAAACAACCAAG | Couple 4 | 345 |
|  | GP_CCHF_seg S_1375R | AGGTGTTCAGAGGCTACAATG |  |  |
|  | GP_CCHF_seg S_1448S | GTCAAGGGCAATGCCACCAG | Couple 5 | 258 |
|  | GP_CCHF_seg S_1706R | TCTCAAAGATATCGTTGCCGCA |  |  |
|  | GP_CCHF_seg S_1689R | CGCACAGCCCTTTAAGTRTTT |  |  |
|  | CCHF_seg S_111S | TGGACACCTTCACAAACTC | Couple 6 | 1029 |
|  | CCHF_seg S_1140R | GACAATTCCCTACACC |  |  |
| M Segment | GP_CCHF_seg M_1S | TCTCAAAGAAATACTTGCGGCAC | Couple 1 | 1958 |
|  | GP_CCHF_seg M_1959R | CCTGTGGCACTGTTTTCGCA |  |  |
|  | GP_CCHF_seg M_1842S | AACTATGGTGGYCCRGGTGAYA | Couple 2 | 1922 |
|  | GP_CCHF_seg M_3764R | CTCCAGTTTCTTGARTGRGGC |  |  |
|  | GP_CCHF_seg M_3711S | GAYTGCCCGGAAAGRTGTGG | Couple 3 | 1855 |
|  | GP_CCHF_seg M_5566R | TCTCAAAGATATAGTGGCGGC |  |  |
| L Segment | GP_CCHF_seg L_1S | TCTCAAAGATATCAATCCCCCC | Couple 1 | 1488 |
|  | GP_CCHF_seg L_1489R | GAGATCAGCTATCTCCCTGTG |  |  |
|  | GP_CCHF_seg L_1259S | GYAACCTAGGAAATGAACTGTTG | Couple 2 | 1708 |
|  | GP_CCHF_seg L_2967R | CTTTGCGCATWGCCTGTTCC |  |  |
|  | GP_CCHF_seg L_2809S | GTTGTTGGAGCYATAAGTACTC | Couple 3 | 1605 |
|  | GP_CCHF_seg L_4414R | GGARTGATTTTCAATGTCTTG |  |  |
|  | GP_CCHF_seg L_4298S | AGGAGRCAAGCTGTCCTTGG | Couple 4 | 1888 |
|  | GP_CCHF_seg L_6178R | CTTTGACARTTCCAGRTGCTG |  |  |
|  | GP_CCHF_seg L_6022S | CTCAACGAGCAACAAGATGAAC | Couple5 | 1584 |
|  | GP_CCHF_seg L_7606R | CATGYTCAACATGTACTGTTGTC |  |  |
|  | GP_CCHF_seg L_7491S | GYTACAACCATATGGGTCAGG | Couple 6 | 1808 |
|  | GP_CCHF_seg L_9299R | GCAGGTCTAGACTCAACTATTC |  |  |
|  | GP_CCHF_seg L_9077S | CTCACTGGTTGGACACCTTTC | Couple 7 | 1547 |
|  | GP_CCHF_seg L_10624R | CTRTCTGTAGAGCAGTCMAC |  |  |
|  | GP_CCHF_seg L_10253S | GTGARACTGAAAGRCAAGTGC | Couple 8 | 1958 |

Supplementary Table S2 : List of GenBank accession numbers for the sequences generated in this study (https://www.ncbi.nlm.nih.gov/genbank).

| **Sample ID** | **Segment** | **Accession number** |
| --- | --- | --- |
| 3154 | L | PV976879 |
| 2456 | L | PV976880 |
| 1002 | L | PV976881 |
| 590 | L | PV976882 |
| 2456 | M | PV976877 |
| 3154 | M | PV976878 |
| 1002 | M | PV976883 |
| 590 | M | PV976884 |
| 3424 | S | PV976885 |
| 3445 | S | PV976886 |
| 590 | S | PV976887 |
| 761 | S | PV976888 |
| 2005 | S | PV976889 |
| 2150_2151 | S | PV976890 |
| 743 | S | PV976891 |
| 1002 | S | PV976892 |
| 1008 | S | PV976893 |
| 2456 | S | PV976894 |
| 3154 | S | PV976895 |
| 2425 | S | PV976896 |
| 2447 | S | PV976897 |

Supplementary Table S3 : Distribution and comparison of demographic and geographic characteristics according to serological status in cattle.

|  | **Negative,**  **N = 510*^1^*** | **Positive,**  **N = 47*^1^*** | **Overall,**  **N = 557*^1^*** | **p-value*^2^*** |
| --- | --- | --- | --- | --- |
| **Age (months)** | Moy. : 14  [min-max : 2 - 217] | Moy. : 22  [min-max : 2 - 229] | Moy. : 15  [min-max : 2 - 229] | 0.4 |
| **Sex** |  |  |  | 0.2 |
| *M* | 316 (62%) | 26 (55.3%) | 342 (61.4%) |  |
| *F* | 194 (38%) | 21 (44.7%) | 215 (38.6%) |  |
| **Age groups** |  |  |  | 0.3 |
| *< 1 year old* | 431 (84.5%) | 38 (80.9%) | 469 (84.2%) |  |
| *> 1 year old* | 79 (15.5%) | 9 (19.1%) | 88 (15.8%) |  |
| **Localisation** |  |  |  | 0.5 |
| *Calvi* | 29 (5.7%) | 2 (4.2%) | 31 (5.6%) |  |
| *Bastia* | 11 (2.2%) | 0 (0%) | 11 (2.0%) |  |
| *Ajaccio* | 300 (58.7%) | 31 (66%) | 331 (59.4%) |  |
| *Sartène* | 109 (21.4%) | 6 (12.8%) | 115 (20.6%) |  |
| *Corte* | 61 (12%) | 8 (17%) | 69 (12.4%) |  |
|  |  |  |  |  |

*^1^ Moy. : Mean [min-max : Range]; n (%)
^2^ Wilcoxon rank sum test; Pearson’s Chi-squared test; Fisher’s exact test*

Supplementary Table S4 : Supplementary Table S3: Distribution and comparison of demographic and geographic characteristics according to CCHFV RNA status in cattle. **Note:** The 11 cattle for which characteristics could not be determined were excluded from the analyses and are therefore not shown in the table.

|  | **Negative,**  **N = 1,054*^1^*** | **Positive,**  **N = 31*^1^*** | **Overall,**  **N = 1,085*^1^*** | **p-value*^2^*** |
| --- | --- | --- | --- | --- |
| **Age (months)** | Moy. : 16 [min-max : 2 - 337] | Moy. : 19 [min-max : 3 - 229] | Moy. : 16 [min-max : 2 - 337] | 0.6 |
| **Sex** |  |  |  | 0.5 |
| *M* | 653 (62%) | 21 (68%) | 674 (62%) |  |
| *F* | 401 (38%) | 10 (32%) | 411 (38%) |  |
| **Age groups** |  |  |  | >0.9 |
| *< 1 year old* | 847 (80%) | 25 (81%) | 872 (80%) |  |
| *> 1 year old* | 207 (20%) | 6 (19%) | 213 (20%) |  |
| **Localisation** |  |  |  | 0.3 |
| *Sartène* | 126 (12%) | 3 (9.7%) | 129 (12%) |  |
| *Bastia* | 33 (3.1%) | 0 (0%) | 33 (3.0%) |  |
| *Ajaccio* | 369 (35%) | 15 (48%) | 384 (35%) |  |
| *Calvi* | 179 (17%) | 7 (23%) | 186 (17%) |  |
| *Corte* | 347 (33%) | 6 (19%) | 353 (33%) |  |
|  |  |  |  |  |

*^1^ Moy. : Mean [min-max : Range]; n (%)
^2^ Wilcoxon rank sum test; Pearson’s Chi-squared test; Fisher’s exact test*

Supplementary Table S5 : Individual cattle sampling data and IgG prevalence by municipality.

|  |  |  |  |  |  |
| --- | --- | --- | --- | --- | --- |
| **Sampling date** | **Cattle ID** | **Cattle age (months)** | **Cattle sex** | **Municipality** | **IgG prevalence (%)** |
| 11/6/2024 | 2030897440 | 7 | M | Alata | 0,90 |
| 11/6/2024 | 2030897441 | 7 | M | Alata |  |
| 5/11/2024 | 2030897462 | 6 | M | Alata |  |
| 9/7/2024 | 2030903556 | 8 | F | Alata |  |
| 10/12/2024 | 2030903574 | 2 | M | Alata |  |
| 14/5/2024 | 2030880433 | 26 | F | Ambiegna | 0,18 |
| 9/7/2024 | 2030786724 | 169 | F | Appietto | 0,36 |
| 19/3/2024 | 2030896596 | 8 | F | Appietto |  |
| 21/1/2025 | 2030898917 | 9 | F | Arro | 0,18 |
| 10/12/2024 | 2030903058 | 17 | M | Bastelica | 0,18 |
| 19/3/2024 | 2030893670 | 10 | M | Bilia | 0,18 |
| 2/4/2024 | 2030875622 | 13 | M | Bocognano | 0,18 |
| 19/11/2024 | 2030911576 | 9 | F | Calcatoggio | 0,18 |
| 10/12/2024 | 2005373936 | 6 | M | Canavaggia | 0,36 |
| 10/12/2024 | 2005373943 | 5 | M | Canavaggia |  |
| 25/6/2024 | 2030710211 | 229 | F | Cargèse | 1,26 |
| 21/1/2025 | 2030897200 | 10 | F | Cargèse |  |
| 4/6/2024 | 2030902784 | 7 | F | Cargèse |  |
| 24/9/2024 | 2030902812 | 7 | M | Cargèse |  |
| 23/7/2024 | 2030902818 | 4 | M | Cargèse |  |
| 10/12/2024 | 2030906654* | 7 | M | Cargèse |  |
| 22/10/2024 | 2030902787* | 7 | F | Cargèse |  |
| 19/11/2024 | 2030894232 | 9 | M | Casaglione | 0,36 |
| 21/1/2025 | 2030909417 | 10 | M | Casaglione |  |
| 21/1/2025 | 2005369733 | 8 | F | Castello-di-Rostino | 0,18 |
| 19/3/2024 | 2030898141 | 8 | M | Ciamannacce | 0,18 |
| 11/6/2024 | 2030897591 | 5 | M | Cozzano | 0,18 |
| 16/4/2024 | 2030012791 | 138 | F | Cuttoli-Corticchiato | 0,90 |
| 25/6/2024 | 2030893059 | 14 | M | Cuttoli-Corticchiato |  |
| 25/6/2024 | 2030893060 | 14 | F | Cuttoli-Corticchiato |  |
| 25/6/2024 | 2030893061 | 4 | M | Cuttoli-Corticchiato |  |
| 9/7/2024 | 2030900641 | 4 | M | Cuttoli-Corticchiato |  |
| 19/3/2024 | 2030900924 | 11 | M | Guitera-les-Bains | 0,18 |
| 2/4/2024 | 2030896396 | 10 | M | Partinello | 0,18 |
| 24/9/2024 | 2005374179 | 6 | M | Lozzi | 0,54 |
| 24/9/2024 | 2005374186 | 5 | M | Lozzi |  |
| 24/9/2024 | 2005380367 | 3 | M | Lozzi |  |
| 5/11/2024 | 2030900906 | 7 | F | Petreto-Bicchisano | 0,36 |
| 4/6/2024 | 2030903811 | 7 | F | Petreto-Bicchisano |  |
| 16/4/2024 | 2005366965 | 6 | F | Pietralba | 0,36 |
| 7/1/2025 | 2005367674 | 6 | M | Pietralba |  |
| 3/9/2024 | 2030897976 | 8 | F | Sartène | 0,36 |
| 8/10/2024 | 2030907226 | 5 | F | Sartène |  |
| 11/6/2024 | 2030897018 | 7 | F | Sotta | 0,18 |
| 27/2/2024 | 2005374150 | 7 | F | Ventiseri | 0,36 |
| 27/2/2024 | 2005374148 | 7 | M | Ventiseri |  |
| 30/4/2024 | 2030895460 | 14 | F | Zigliara | 0,18 |
|  |  |  |  |  |  |

* Animal where tick pools were tested positives to CCHFV RNA
